# Retinoic Acid (RA): A Critical Immunoregulatory Molecule in Asthma and Allergies

**DOI:** 10.1002/iid3.70051

**Published:** 2024-10-28

**Authors:** Ramin Lotfi

**Affiliations:** ^1^ Blood Transfusion Research Center, High Institute for Research and Education in Transfusion Medicine Tehran Iran; ^2^ Kurdistan Regional Blood Transfusion Center Sanandaj Iran; ^3^ Clinical Research Development Center, Tohid Hospital Kurdistan University of Medical Sciences Sanandaj Iran; ^4^ Lung Diseases and Allergy Research Center, Research Institute for Health Development Kurdistan University of Medical Sciences Sanandaj Iran

**Keywords:** allergy, asthma, retinoic acid, tolerance, vitamin A

## Abstract

**Introduction:**

Asthma and allergies are chronic inflammatory disorders that are triggered owing to aberrant responses of the immune system against typically innocent environmental substances. Retinoic acid (RA) represents a biologically active metabolite of vitamin A (VA) and high‐affinity ligand for RA receptor (RAR) that is implicated in a wide variety of biological processes, including cell proliferation, differentiation, apoptosis, organogenesis, reproduction, and immune responses. In the immune system, RA contributes to the induction of regulatory T (Treg) cells, adhesion molecules required for homing of B and T cells in the gut, and tolerance. Noteworthy, RA has a pivotal role in maintaining the balance of Th17‐Treg cells and is also indispensable for appropriate responses of T helper (Th) cells.

**Aims:**

This mini‐review article intends to expose the immune functions of RA, with an emphasis on the enzymatic pathways converting VA into RA and its receptor‐dependent actions in asthma and allergies.

**Conclusions:**

Recent findings have depicted that RA levels are reduced in asthma and allergies and that treatment with RA alleviates allergy symptoms and airway inflammation. RA also modulates allergic airway disorders by inhibiting Th2/Th17 response and increasing Treg cells. Therefore, RA could be considered a novel and promising therapeutic agent to be studied and used for treating these diseases.

## Summary

- Retinoic acid (RA) is indispensable for appropriate responses of T helper (Th) cells.
- RA has a pivotal role in maintaining the balance of Th17‐Treg cells.
- RA levels are reduced in asthma and allergies.
- Treatment with RA alleviates allergy symptoms and airway inflammation.
- RA emerges as a novel and promising therapeutic agent to treat asthma and allergies.

## Graphical abstract

RA has a pivotal role in maintaining the balance of Th17‐Treg cells and is also indispensable for appropriate responses of T helper (Th) cells. Recent evidence has depicted that RA levels are reduced in asthma and allergies and that treatment with RA alleviates allergy symptoms and airway inflammation. Therefore, RA could be considered a novel and promising therapeutic agent to be studied and used for treating asthma and allergies.

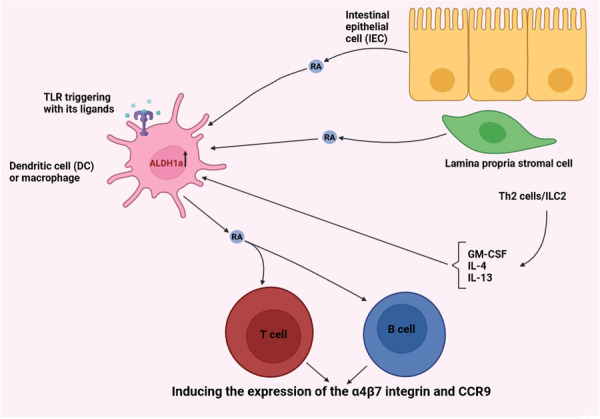

## 1 Introduction

The prevalence of asthma and allergic diseases (allergies) has rapidly increased over the recent decades in both developed and developing countries, now becoming a public health problem around the world [1, 2]. Asthma and allergies are chronic inflammatory disorders developed in genetically prone persons due to the inappropriate responses of the immune system against foreign, usually innocuous, substances [2, 3].

Genetic factors, immune system, as well as changing lifestyle and/or environment are the most identified risk factors in the development of these diseases. The alterations in dietary patterns may particularly exert a key role. For instance, decreased intake of the antioxidant vitamins A, C, and E may be related to the increased prevalence of asthma and allergic diseases [4, 5, 6]. In this regard, the intake of dietary vitamin A (VA) and serum levels of VA are strikingly decreased in asthmatic patients compared with healthy people or in patients having severe asthma relative to mild asthma. Epidemiological investigations have also found an inverse relationship between asthma and the intake of VA [7, 8, 9, 10]. Other studies; however, have achieved no such relationships between VA and allergic rhinitis (AR) [11, 12]. In a murine model of allergic airway inflammation with high levels of immunoglobulin E (IgE) and IgG1 antibodies, it was found that high dietary intake of VA intensifies the severity of experimental asthma and results in T helper 2 (Th2) skewing, which in turn may cause asthma exacerbation. Likewise, this study revealed that VA deficiency decreases serum IgE levels, pulmonary eosinophilia and hyperresponsiveness, and the levels of interleukin 4 (IL‐4) and IL‐5 cytokines in bronchoalveolar lavage (BAL) specimens, by promoting the responses toward Th1 cells [13]. On the other hand, VA deficiency can lead to increased mortality and adverse outcomes like blindness, especially in children [14]. VA deficiency was also associated with impaired intestinal immune responses [15, 16] and enhanced mortality related to gastrointestinal and respiratory infections [17]. On the contrary, VA supplementation was associated with a striking reduction in diarrhea and mortality in malnourished children [18, 19]. Thus, the relationship between VA and asthma/allergic diseases is contradictory and not completely clear [11, 12]. This contradiction may be due to the dualistic roles of VA and its derivative (i.e., RA), local microenvironment, local VA/RA levels, additional cytokines, and other microenvironmental factors that may affect asthma/allergies [20].

All‐trans retinoic acid (ATRA) is a biologically active metabolite of vitamin A, playing a broad spectrum of important bioactions in cell proliferation, differentiation, apoptosis, organogenesis, reproduction, and immune responses [14, 19, 21]. The biological actions of RA are exerted through binding to its receptors located at the nucleus, termed retinoic acid receptors (RAR), and this interaction leads to promoting the transcription of multiple target genes [10]. In the intestinal mucosa; moreover, RA as a regulatory signal exhibits anti‐inflammatory actions [14] and acts to augment the differentiation of forkhead box P3 (FoxP3)^+^ regulatory T (Treg) cells and produce IgA from B cells [22, 23]. In an animal model of rheumatoid arthritis, that is, collagen‐induced arthritis, ATRA treatment also inhibits Th17 cell differentiation while boosting Treg cell differentiation, thereby alleviating intestinal inflammation, synovial inflammation, and arthritis [24]. Besides, RA was found to influence various immune cells, comprising B cells and their antibody responses, T cells count, homing, and their skewing to different subsets [25, 26, 27, 28].

Previously, studies have illustrated that both VA and RA skew the responses toward Th2 cells and, thereby, raise the ratio of Th2/Th1 cytokines production by decreasing the Th1 responses [29, 30]. Also, RA inhibits the Th17 cells and boosts the Treg cells that are critical for establishing and retaining immune homeostasis, equilibrating immunity, and tolerance [4, 14, 31]. By contrast, RA was reported to decrease airway inflammation by suppressing the differentiation and/or functions of Th2 cells [10]. This mini‐review article aims to clarify in more detail the immune functions of RA, focusing on the enzymatic pathways converting VA into RA and its receptor‐dependent functions in asthma and allergies.

## 2 The Metabolism of Vitamin A (VA)

**Figure 1:**
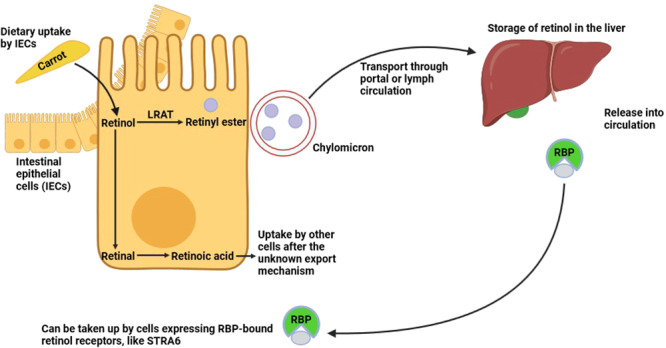
The metabolism of vitamin A (VA). Diet‐derived retinol is absorbed from the intestinal lumen by intestinal epithelial cells (IECs), cells that are recognized not only to be involved in the metabolization of retinol into retinoic acid (RA) but can also transport retinol further towards for instance the liver. In addition to IECs, RA can also be generated by lamina propria stromal cells. Retinol is afterward either metabolized into RA and exported through unknown mechanisms or converted into its storage form, that is, retinyl esters and transported, packaged in chylomicrons form, to be stored in the liver. During homeostasis in the liver, retinol can be released into the general circulation bound by retinol‐binding protein (RBP), and RBP‐bound retinol can be absorbed by cells expressing RBP‐bound retinol receptors. The figure was made using BioRender.

VA is a fat‐soluble nutrient that is indispensable for body health, as well as the proper function of the immune system. It consists of retinol and over 600 carotenoids, including β‐carotene, β‐cryptoxanthin, lutein‐zeaxan‐thin, and lycopene [4]. Noteworthy, ATRA plays a predominant regulatory role in the immune system among the various VA metabolites. VA is acquired from the diet via the consumption of plant food (e.g., carrots, red peppers, lettuce) and animal food (e.g., fish, liver, milk, eggs) sources, which respectively contain its precursors such as carotenoids and VA in the form of retinol or retinyl esters [32, 33, 34]. In the intestines, the intestinal epithelial cells (IECs) absorb diet‐derived VA or its precursors. Using the lecithin retinol acyltransferase (LRAT) enzyme; afterward, they are esterified into retinyl esters form that can be packed into chylomicrons by IECs and are subsequently transported to the lymphatic system or the portal vein [20]. In the systemic circulation, the enzymatic action of lipoprotein lipase (LPL) on the chylomicrons leads to their capture by hepatocytes and hydrolysis to retinol, which is absorbed by the liver, an organ in which the biggest amounts of retinol are stored, mainly in hepatic stellate cells (HSCs) [19]. Also, IECs can convert the diet‐derived VA or its precursors into retinal and RA, which can directly be liberated in the intestinal microenvironment through the unknown export mechanism. Subsequently, retinal and RA are taken up by other cells [35]. Importantly, retinol bound with either retinol‐binding protein (RBP) or serum amyloid A (SAA) depending on the homeostasis or infection status (respectively) is entered again into the bloodstream (Figure 1) [36, 37].

**Figure 2:**
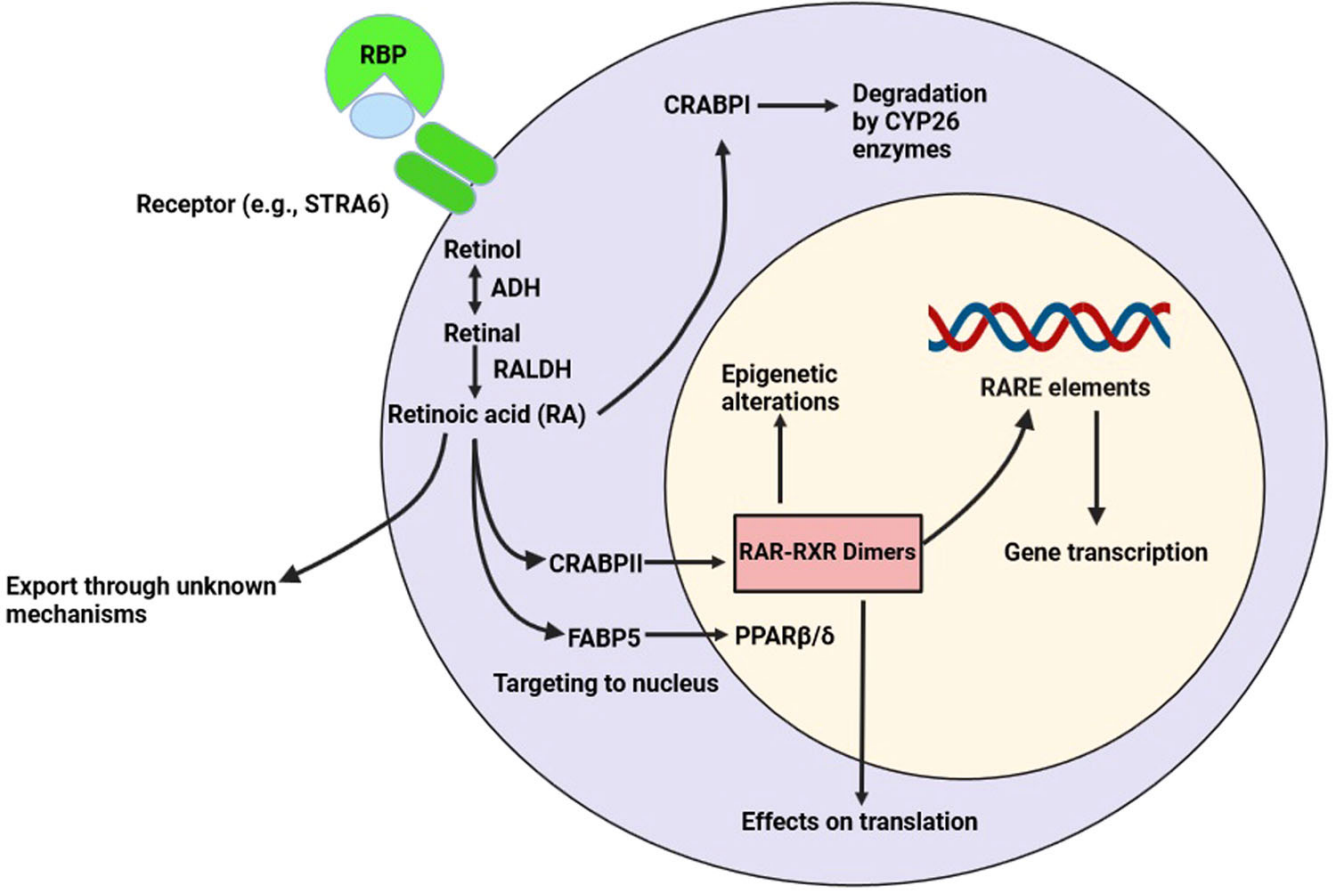
The signaling of retinoic acid (RA). Retinol bound to retinol‐binding protein (RBP) can be absorbed by cells expressing the stimulated by retinoic acid 6 receptor (STRA6) receptor or additional unknown receptors. Using the enzymatic actions of alcohol dehydrogenase (ADH) and retinaldehyde dehydrogenase (RALDH) enzymes, retinol is converted into RA within the cytosol, where it can either be transported to the nucleus using the cellular RA‐binding protein (CRABP) II and fatty‐acid binding protein 5 (FABP5) enzymes or targeted for degradation through the CRABPI enzyme. Within the nucleus, upon RA connecting to its receptors, that is, retinoic acid receptor (RAR: having three isoforms of α, β, and γ), retinoid X receptor (RXR: possessing three isoforms of α, β, and γ), or peroxisome proliferator‐activated receptor (PPAR) β/δ, these nuclear receptors can now function as transcription factors by connecting to RA response elements (RARE) in target genes or can affect translation or epigenetics. The figure was made using BioRender.

Indeed, retinol along with RBP forms a complex and is continuously transported into the bloodstream through the liver [38]. This complex is identified via the stimulated retinoic acid 6 (STRA6) receptor mediating the absorption of retinol to the cytosol (Figure 2) [39, 40]. Nonetheless, the STRA6 receptor is merely necessary for retaining RA homeostasis in the eye; hence, additional mechanisms presumably are involved in uptaking the retinol into other tissues [40, 41]. Following the uptake of retinol to the cell cytosol, it is oxidized to the retinal form (also known as retinaldehyde) by a reversible enzymatic reaction mediated by alcohol dehydrogenase (ADH), which is ubiquitously expressed in the cell [32]. Depending on the cell type such as IECs, dendritic cells (DCs), and macrophages related to mesenteric lymph nodes and Peyer's patches; then, the retinal can irreversibly be converted into RA by the enzymatic activity of retinaldehyde dehydrogenase (RALDH: also known as aldehyde dehydrogenase; ALDH) (Figure 2) [19]. There are many isoforms of the RALDH enzyme, of which three isoforms, namely, RALDH1 (ALDH1a1), RALDH2 (ALDH1a2), and RALDH3 (ALDH1a3), are widely studied in cells of the immune system [20, 42]. Besides, RA was recently found to be biosynthesized through an alternative pathway involving the ALDH3b1 enzyme in mouse macrophages and bone marrow cells [43]. Because RALDH is considered the predominant enzyme that determines the cell populations producing RA [32], controlling the expression of different isoforms for this enzyme can regulate RA levels and their expression in various cells and tissues [42].

## 3 The Signaling of Retinoic Acid (RA)

In the human body, all‐trans RA (ATRA) is typically regarded as the most common biological form of RA, which occurs in at least six isomers [44, 45]. Within the cytosol, RA can interact with three binding proteins, including cellular retinoic acid‐binding protein 1 (CRABPI), CRABPII, and fatty acid‐binding protein 5 (FABP5). Connecting RA to CRABPI causes RA to be degraded by enzymes of the cytochrome P450 family 26 (CYP26), which belongs to the cytochrome P450 enzyme (CYP) superfamily. Amongst the members of the CYP26 family, CYP26 subfamily A member 1 (CYP26A1) and CYP26B1 are the most important members with high catalytic efficiency to perform this function [46]. Indeed, tissue and cellular concentrations of RA are overall regulated via homeostatic processes inclusive of both RA generation and degradation, by using the CYP26 enzymes (i.e., CYP26A1, CYP26B1, and CYP26C1) mainly mediating the catalysis of RA to inactive products, like 4‐oxo RA and 4‐hydroxy RA [47, 48]. Noteworthy, these enzymes function to prevent RA agglomeration in the body and to retain optimal physiologic concentrations of RA for the best efficiency [14].

Noteworthy, upon RA binding to CRABPII or FABP5, they translocate to the nucleus. Within the nucleus, CRABPII‐bound RA is aimed at the RAR receptors, while FABP5‐bound RA is aimed at the peroxisome proliferator‐activated receptor‐β/δ (PPAR‐β; also known as PPAR‐δ) [49, 50]. Due to a higher binding affinity of CRABPII for RA than FABP5, RAR seems to be the major signaling pathway induced by RA [49]. Within the nucleus, RA can bind to its receptors, including RARs, retinoid X receptors (RXRs), and PPAR‐β/δ, and then activate them. Connecting RA to RAR can lead to the dimerization of RAR with RXR, which enables the receptor to interact with the retinoic acid response elements (RAREs) located in the DNA, thereby inducing the gene expression. In this route, RA is able to modulate transcription, whereas RAR‐bound RA can besides affect translation and function as a regulator of epigenetic alterations (Figure 2) [51, 52, 53].

## 4 The Role of RA in Orchestrating T Cell Responses

**Figure 3:**
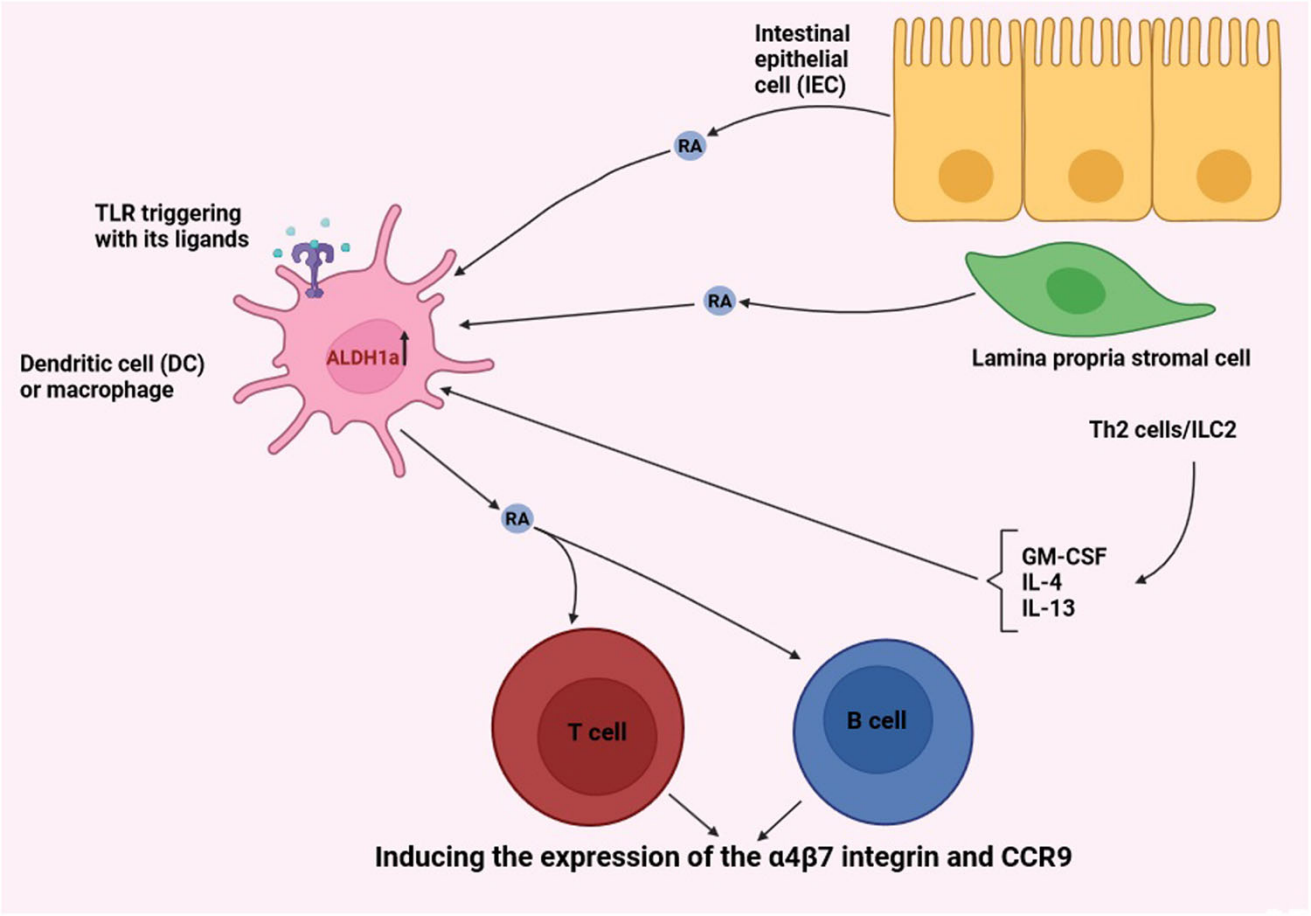
RA metabolism in DCs and macrophages and its role in instructing B and T cells. Both lamina propria stromal cells and IECs generate RA, which is the most significant inducer of the RA‐generating enzyme, that is, ALDH1a, in DCs and macrophages. By generating granulocyte‐macrophage colony‐stimulating factor (GM‐CSF), the lamina propria stromal cells can induce ALDH1a expression in DCs and macrophages. Similar to GM‐CSF, the cytokines of IL‐4 and IL‐13 are also found to be able to induce ALDH1a expression in DCs and macrophages. Type 2 innate lymphoid cells (ILC2) and Th2 cells are known to be the main sources of producing the mentioned cytokines. Besides cytokines, the engagement of the toll‐like receptor 2 (TLR2) and TLR5 with their associated ligands can also induce ALDH1a expression in DCs. Indeed, ALDH1a expression is necessary for producing RA by DCs and macrophages to train T and B cells with RA and to induce the expression of α4β7 integrin and CCR9 on B and T cells. The figure was made using BioRender.

Macrophages and DCs are the predominant sources of RA for B and T cells and via producing RA, can induce the expression of the gut‐homing adhesion molecules, including α4β7 integrin (also referred to as lymphocyte Peyer patch adhesion molecule; LPAM) and C‐C chemokine receptor 9 (CCR9) on B and T cells (Figure 3) [54, 55]. RA also possesses a supplementary role in the induction of effector functions of T cells, and it can excite inflammatory or tolerogenic effects depending on the milieu and synergizing cytokines to which cells are encountered [20]. In homeostatic circumstances, RA generated by DCs or macrophages, along with transforming growth factor‐β (TGF‐β) cytokine, induces naïve T cells to be converted to Treg cells at the expense of inhibiting Th17 cells. Along with TGF‐β, RA also augments the expression of FoxP3, thereby further provoking Treg cell differentiation. Moreover, RA boosts the generation of the anti‐inflammatory cytokine interleukin‐10 (IL‐10) in Treg cells [20, 56]. Simultaneously, RA inhibits the signaling of IL‐6 to hinder the naïve T cells conversion into Th17 cells, and along with IL‐2, it also restrains the Treg cells conversion into Th17 cells. Besides, RA inhibits the IL‐21, IL‐4, and interferon‐γ (IFN‐γ) generation by CD4^+^CD44^high^ T cells which support inducing a Th17 cell phenotype [57]. In inflammatory circumstances, RA along with IL‐15 is able to promote the generation of IL‐23 and IL‐12 by DCs. The mentioned cytokines, afterward, act to exert a key role in increasing Th17 and Th1 subsets, respectively. According to evidence, RA effects seem to be dose‐dependent so that at low levels, it can boost the differentiation of Th17 cells, but at higher levels, as reported in the gut, supports the induction of Treg cells. From the above‐mentioned explanations, it can be inferred that RA is a pivotal ascertaining molecule in the maintenance of the balancing of Th17 and Treg cells. Additionally, RA increases the signaling of IL‐12 and IFN‐γ in the induction of Th1 cells, and IL‐4 signaling in inducing Th2 cells [20, 58].

## 5 RA in Asthma and Allergies

Allergic reactions are inflammatory states caused by a variety of factors, including external and internal factors. External factors comprise alterations in gut microbiota and metabolites, air pollution, and drugs. Internal factors encompass genetic and epigenetic alterations [59]. Allergic diseases can be initiated by imbalances between pro‐inflammatory Th2 cells (producing IL‐4, IL‐5, and IL‐13 cytokines) and anti‐inflammatory Th1 cells, leading to hyperactive immune responses. B cells generate IgE antibodies that identify and connect to allergens (e.g., airborne, pollen, foods, drugs), causing basophils, eosinophils, and mast cells to be activated. The mentioned cells then liberate inflammatory mediators, like histamine and leukotrienes, that cause developing allergy symptoms such as itching, swelling, and difficulty breathing. The combination of these immune responses can result in allergic disorders and asthma, a chronic respiratory condition denoted by airway inflammation, airway hyperresponsiveness (AHR), and narrowing [60]. According to evidence, VA and RA boost differentiation into Th2 cells and the ratio of Th2 cytokines/Th1 cytokines by diminishing the Th1 response. Besides, RA inhibits Th17 cells and augments Treg cells [31]. Indeed, RA promotes Th2 cell differentiation by inducing the expression of the *IL‐4* gene [61]. Furthermore, RA inhibits the expression of the master regulator transcription factor of Th1 cells, that is, T‐bet, and induces the transcription factors promoting Th2 cells, such as GATA‐binding protein 3 (GATA3), macrophage‐activating factor (MAF), and signal transducer and activator of transcription 6 (STAT6) [29, 62]. RA has been suggested to indirectly fulfill its Th2 cell‐inducing effect via the modulation of APCs [63]. Nevertheless, RA is also able to function directly on T cells to promote the differentiation of Th2 cells via RAR proteins [29, 62]. A newly published meta‐analysis in 2023 indicated that serum concentrations of RA precursor, that is, VA, are diminished in patients with asthma when compared to healthy persons. It was also revealed that a relatively higher intake of VA during pregnancy is linked to an enhanced risk of asthma at age 7 years [64]. RA and retinol levels were reported to be significantly decreased in lung tissues obtained from asthmatic patients and house dust mite (HDM)‐challenged mice, in comparison to non‐asthmatic human lungs and phosphate buffer saline (PBS)‐challenged mice, in order. The findings of this study propose that the metabolism of RA is diminished in asthmatic lungs and that increasing the signaling of RAR by using ATRA or RARγ agonists may alleviate asthma‐related airway remodeling [65]. Recently, RA serum concentrations were found to be diminished in allergic rhinitis (AR) patients than in healthy subjects; however, their difference was not statistically meaningful [66]. Moreover, RA concentrations were positively correlated with the levels of IL‐10 and TGF‐β in the sera of AR patients [67]. In a murine model with food allergy, it was indicated that the continuous intake of RA under allergen exposure improves the intensity of food allergy [68]. Additionally, intraperitoneal (I.P.) administration of ATRA in an AR murine model strikingly reduced allergic symptom scores in the ATRA group when compared to the AR group. Also, the serum concentrations of *Dermatophagoides farina*‐specific immunoglobulin E (IgE), as well as nasal mucosa infiltration of eosinophils and their count, were diminished in the ATRA group relative to the AR group. While the mRNA expression level of IFN‐γ was higher, the relative expression level of IL‐4, GATA‐3, and RORγt mRNA was lower in the nasal mucosa of the ATRA group compared to the AR group. In addition, the mRNA expression level of FoxP3, IL‐10, and TGF‐β, as well as the percentage of CD4^+^CD25^+^FoxP3^+^ T cells was considerably increased in the ATRA group relative to the AR group. Western blot analysis results showed similar expression patterns of all measured factors, except for FoxP3, to those of real‐time PCR analyses [4]. Thus, the mechanism by which ATRA exerts the anti‐allergic effects is accomplished by inducing Treg cells and inhibiting Th2 responses. In line with these findings, another study by Fang, Jin, & Wang (2004) in a rat model of asthma demonstrated that the total cell counts and inflammatory cell proportion in bronchoalveolar lavage fluid (BALF) are lower in the ATRA group than in the asthma group. Also, the pulmonary pathological changes were attenuated in the ATRA group compared to the asthma group. The protein expression of nuclear factor‐kappa B (NF‐κB) inhibitor (IκBa) was increased in the ATRA group than asthma group, whereas the expression of NF‐κB and intercellular adhesion molecule 1 (ICAM‐1) was reduced in the ATRA group relative to the asthma group [69]. This study revealed the anti‐inflammatory effects of ATRA on airway inflammation in asthma. Also, it was found that the anti‐inflammatory effects of ATRA are similar to dexamethasone effects. These data generally indicate that ATRA actions are exerted through enhancing the cytoplasmic IκBa content and subsequently suppressing the NF‐κB activation and expression. Besides that, the study of Wu et al. (2013) showed that treatment with ATRA in an allergic airway inflammation murine model attenuates airway inflammation and reduces the mRNA expression level of Th2‐ and Th17‐associated transcription factors (GATA‐3 and RORγt, respectively) and their cytokines levels (IL‐4 and IL‐17, respectively) [10].

Concurrent injection of ATRA with OVA orally into a mouse model of bronchial asthma caused striking reductions in AHR, airway eosinophilia, elevated levels of cytokines in BALF, and metaplasia of goblet cells. In contrast, the proportion of Treg cells in the lungs of mice that underwent OVA and ATRA treatment was enhanced, in comparison with those treated with OVA only. In addition, transferring the pulmonary CD4^+^ T cells isolated from OVA and ATRA‐treated mice caused suppression of AHR and airway inflammation. In this study, when the antagonist of the RAR, that is, the LE135, was utilized, the effects of ATRA on AHR, allergic airway inflammation, and the count of Treg cells in the lungs were entirely inverted [70]. In a mouse airway allergy model, therapy with ATRA diminished inflammation and Th17 cell‐associated cytokines whereas Treg cell count in the lung was increased [10, 70, 71]. Nonetheless, this enhancement of Treg cells was time‐dependent, and the useful effects of ATRA in asthma may be fading due to the increased eosinophilia that also happened upon ATRA treatment [13, 71]. In aggregation, these findings show that RA has anti‐allergic effects and can be applied as a therapeutic agent for treating asthma and allergies in the coming years. The therapeutic effects of RA on asthma and allergies are summarized in Table 1.

**Table 1:** The beneficial effects of RA on asthma and allergies.

Condition	Site	Expression changes	Role	Reference (s)
**AR patients**	Serum	Decreased, but not significant RA was positively correlated with serum TGF‐β and IL‐10 levels	RA may influence AR risk by affecting the TGF‐β and IL‐10 generation	[66, 67]
**Asthmatic patients**	Lung tissues	Reduced	RA metabolism is decreased in asthmatic lungs	[65]
**HDM‐challenged mice**				
**Molecule**	**Model type**	**Injection mode**	**Effects**	**Reference (s)**
**RA**	FA murine model	Oral	Improves the severity of food allergy	[68]
**ATRA**	AR murine model	I.P.	Reduces allergy symptom scores and nasal mucosa infiltration of eosinophils and their count Induces Treg cells and inhibits Th2 responses	[4]
**ATRA**	Asthma rat model	I.P.	Decreases total cell counts and inflammatory cell proportion in BALF Attenuates the pulmonary pathological changes Increases the cytoplasmic IκBa content and subsequently suppresses the NF‐κB activation and expression	[69]
**ATRA**	Experimental allergic asthma (murine model)	I.P.	Attenuates airway inflammation and reduces the mRNA expression level of Th2‐ and Th17‐associated transcription factors and their cytokines levels	[10]
**ATRA**	Bronchial asthma mouse model	Oral	Decreases AHR, airway eosinophilia, elevated levels of cytokines in BALF, and metaplasia of goblet cells Enhances the proportion of Treg cells in the lungs	[70]

Abbreviations: AHR, airway hyperresponsiveness; AR, allergic rhinitis; ATRA, all‐trans retinoic acid; BALF, bronchoalveolar lavage fluid; FA, food allergy; HDM, house dust mite; IκBa, nuclear factor‐kappa B inhibitor; IL‐10, interleukin‐10; I.P., intraperitoneal; NF‐κB, nuclear factor‐kappa B; RA, retinoic acid; Treg cell, regulatory T cell; TGF‐β, transforming growth factor‐β; Th2, T helper 2.

## 6 RA Receptors and Signaling

RA exerts its bioactions primarily through interaction with the nuclear receptors of the RAR family, which belongs to the nuclear hormone receptors (NHR) superfamily of transcription factors. The RAR family has three major isoforms, including RARα, RARβ, and RARγ. These form heterodimers with the members of the retinoid X receptor (RXR) subfamily, namely, RXRα, RXRβ, and RXRγ, thereby acting as ligand‐dependent transcriptional regulators by interacting with RA response elements (RAREs) located in the promoters of RA‐responsive genes [19, 72, 73]. Besides, RA can bind to the PPARβ/δ nuclear receptor when it heterodimerizes with RXR, which this signaling pathway is probably important for lipid metabolism and glucose homeostasis [49]. The ratio of CRABPs to FABP5 determines whether RA signaling occurs via RAR or PPARβ/δ, which translates as distinct functional consequences [49]. RA also has low‐affinity receptors; including chicken ovalbumin upstream promoter transcription factor II (COUP‐TFII) and hepatocyte nuclear factor 4 (HNF‐4) receptors when they form a heterodimer with RXR. Signaling from these receptors is important for lipid metabolism and glucose homeostasis, similar to PPARβ/δ [74, 75].

## 7 Conclusions and Future Perspectives

In summary, RA plays a critical function in regulating immune responses, and evidence has indicated that it possesses a dual role. Numerous factors, including the signaling of TLR, the existence of other cytokines, local concentrations of RA, and the cellular and molecular composition of the microenvironment, ascertain the effector actions of RA. In addition to promoting the transcription of multiple genes, RA can also affect translation or stimulate epigenetic effects by connecting to its nuclear receptors, including RARs, RXRs, and PPAR‐β/δ. As mentioned in this review, several studies have shown the beneficial therapeutic effects of RA in asthma and allergies, introducing RA as a novel and promising therapeutic agent for the treatment of these diseases. Nonetheless, an essential point to keep in mind is that since the effector functions of RA are dependent on the local microenvironment as well as the clinical and immunological condition of the patient, the therapeutic consequence of RA therapy is also anticipated to be varied. Therefore, more investigations would be imperative for further deciphering the interaction of RA with the local microenvironment and how these interactions are adjusted to be able to design efficacious remedies.

## Author Contributions

**Ramin Lotfi:** conceptualization; methodology; validation; writing–original draft; writing–review & editing.

## Conflicts of Interest

There are no conflicts of interest with respect to this manuscript.

## Data Availability

Data sharing does not apply to this article as no datasets were generated or analyzed during the present study.
